# Cardiac Metabolism in Healthy, Senescent and Diseased States

**DOI:** 10.3390/cells15131164

**Published:** 2026-06-26

**Authors:** Uma Bapat, Shahem Albean, Lei Hao, Eun Jung Lee

**Affiliations:** 1 Department of Biological Sciences, New Jersey Institute of Technology, Newark, NJ 07102, USA; uhb2@njit.edu; 2 Department of Biomedical Engineering, New Jersey Institute of Technology, Newark, NJ 07102, USA; sa386@njit.edu (S.A.); lh34@njit.edu (L.H.)

**Keywords:** cardiac metabolism, human-induced pluripotent stem-cell-derived cardiomyocytes (hiPSC-CMs), metabolic maturation, metabolic remodeling, senescence

## Abstract

**Highlights:**

- Dynamic cardiac metabolic shifts are mapped from fetal development to senescence, detailing the distinct bioenergetic profiles of healthy versus diseased states.
- Delineates how cardiac metabolism is regulated by multicellular crosstalk between cardiomyocytes (CMs) and non-CMs.
- The insights bridge stem cell biology with regenerative cardiology, offering strategies to optimize human-induced pluripotent stem-cell-derived cardiomyocyte (hiPSC-CM) models.

**Abstract:**

Cardiovascular disease (CVD) is the leading cause of mortality worldwide. The healthy adult heart depends on flexible energy use, but a diseased or injured heart is associated with a loss of flexibility and metabolic remodeling. Since metabolism plays a central role in cardiac health and disease, there is a growing need to understand how metabolic reprogramming contributes to cardiac dysfunction and impaired CM maturation. Human-induced pluripotent stem-cell-derived cardiomyocytes (hiPSC-CMs) are widely used as a platform to study human cardiac development and disease mechanisms. However, current models are limited by metabolic and structural immaturity. This review provides an overview of the dynamic shifts in cardiac metabolic states from fetal development to senescence, while delineating the metabolic signatures of healthy versus disease states. These metabolic switches are orchestrated by a complex interplay of upstream signals driven by variations in substrate availability, post-translational modifications and key transcriptional regulatory networks, which ultimately regulate downstream cardiac remodeling and pathological cascades. As cardiac metabolic function is affected by a coordinated multicellular network, this review also includes the metabolic crosstalk between CMs and non-CMs, including fibroblasts, endothelial cells and immune cells. In addition, various strategies to further mature hiPSC-CMs are summarized to enhance their metabolic profiles. Investigating cardiac metabolic shifts bridges developmental biology, stem cell biology, and regenerative cardiology by revealing how energy metabolism governs cellular identity, maturation, and regenerative potential. These insights are essential for improving stem-cell-derived CMs for disease modeling, drug discovery, and heart repair.

## 1. Introduction

Cardiovascular disease (CVD) is the number one cause of death worldwide [1], making it an extremely pertinent challenge for society. CVD currently causes 20.5 million deaths worldwide annually and is expected to exceed 23.6 million deaths per year by 2030 [2,3]. Since the heart is one of the most metabolically active organs in the body [4], a better understanding of metabolic remodeling during development through senescence and from healthy to diseased states is critical to prevent and treat related diseases.

A healthy normal heart is dynamic and metabolically flexible. Chemical energy is converted into mechanical energy by multiple metabolic pathways that utilize a variety of energy-providing substrates. These include fatty acids, glucose, pyruvate, ketone bodies, and amino acids depending on availability [5]. In CVD, the heart loses its metabolic flexibility and undergoes metabolic remodeling, which is a shift in what fuels it uses and how efficiently it uses them in response to the diseased environment [6,7,8]. Alterations in fuel substrate preference and shifts in metabolic oxidation rates may occur as a direct consequence of organelle dysfunction within a diseased microenvironment [9,10]. Moreover, loss of metabolic flexibility is usually considered to be associated with impaired metabolic processes and metabolic remodeling plays an important role in the development of CVDs, contributing to heart dysfunction [8]. In the senescing heart, metabolic flexibility is also lost. Cardiac senescence is a key indicator of cardiac damage usually induced by chemotherapy, immunotherapy, or radiotherapy, and can result in conditions of cardiac fibrosis, diastolic dysfunction, arrhythmias, and more [11,12,13]. The process of senescence has multiple facets, including cellular and molecular processes including metabolic reprogramming, mitochondrial dysfunction, and oxidative stress. Accelerated senescence can occur when the balance between lipid synthesis and lipid degradation is disrupted, and intracellular lipid concentration increases [11]. Senescence can also be attributed to elevated cell cycle inhibitor expression, DNA damage, and mitochondrial dysfunction, while causing myocardial stiffness and possibly contributing to progressive heart failure.

Currently, the immaturity of hiPSC-CMs is the major limitation for their use in disease modeling and clinical therapy. HiPSC-CMs maintain a fetal-like metabolic profile characterized by high rates of glycolysis and low rates of fatty-acid oxidation [14]. As metabolic remodeling serves as both a marker and regulator of cardiomyocyte maturation, a better understanding of the transition from glycolytic to oxidative metabolism provides critical insight into stem cell differentiation, cardiac development, and the engineering of functionally mature CMs for regenerative therapies. Thus, this review provides an overview of the dynamic shifts in cardiac metabolic states from fetal development to senescence as well as in disease progression. The metabolic fuel preferences of cardiomyocytes at different developmental and physiological states as well as hiPSC-CMs are summarized in Table 1. It also highlights the metabolic crosstalk between CMs and non-CMs population emphasizing the complex cellular interactions that regulate cardiac function. Moreover, various strategies to enhance metabolic maturation of hiPSC-CMs are discussed and summarized in Table 2. The relevant literature for this review was identified through comprehensive searches of available peer-reviewed publications. Along with recent high-impact review articles, priority was given to primary research articles utilizing human or mammalian models that provided direct mechanistic insights. Studies limited to systemic clinical trial outcomes without direct assessment of cellular, molecular, or tissue-specific metabolic parameters were excluded from consideration. Collectively, understanding the metabolic mechanisms that govern CM development, maturation, and dysfunction is critical for improving the physiological relevance of hiPSC-CM models and advancing translational applications in disease modeling, drug discovery, and regenerative cardiology.

## 2. Metabolism of Healthy Cardiac Cells

### 2.1. Fuel Preference in a Normal Adult CM

In the healthy adult heart, fatty acids, along with carbohydrates, are recognized as the main energy source, accounting for 60–70% of energy [15]. The key metabolites of fatty acids and carbohydrates are fatty acyl-coenzyme A (CoA) and pyruvate. The other 30–40% of energy supplement is derived from glucose [16]. Fatty-acid oxidation produces more energy per molecule compared to glucose oxidation. However, glucose can be metabolized quickly to provide immediate energy, whereas fatty acids serve as a more sustained and long-lasting energy source. For this reason, fatty acids are considered a primary energy source during low-intensity activities. During intensive physical activity, the primary cardiac energy source switches to glucose. Mitochondrial volume occupies approximately 25% of the human CM cytoplasm, approximately four- to tenfold greater than the 3–8% typical of skeletal muscle [17,18]. Mitochondria can utilize both fatty acids and glucose for oxidative metabolism and the entry of these fuels into mitochondrial metabolism is tightly regulated at key rate-limiting steps. In long-chain fatty-acid oxidation, carnitine palmitoyl transferase 1 (CPT-1) regulates the transport of long-chain CoA entering the mitochondria. For glucose, pyruvate dehydrogenase (PDH) controls the conversion of pyruvate into acetyl-CoA, serving as the rate-limiting step of the pyruvate oxidation [19]. Due to the nature of the lipid bilayer of the cell membrane, glucose needs glucose transporters to cross the cell membrane and enter the cell. In the human heart, glucose transporter 4 (GLUT4) accounts for approximately 70% of glucose transporters [20]. Other glucose transporters such as GLUT1, GLUT3, GLUT8, GLUT10, GLUT11, GLUT12 and sodium/glucose cotransporter1 (SGLT1) are also involved in glucose uptake [21].

In addition to fatty acids and glucose, the heart can use a variety of fuels such as lactate, ketone bodies and amino acids based on availability in response to physiological changes [5,15]. This flexibility is largely driven by CMs, which contain a high density of mitochondria to support continuous ATP production through oxidative metabolism [22]. During moderate to intense exercise, CMs undergo marked metabolic adaptation. As skeletal muscles produce large amounts of lactate into the bloodstream, CMs switch to lactate-driven engines to provide 50–60% of total cardiac energy [23]. CMs readily take up this lactate via monocarboxylate transporters (MCT1) [24] and convert it directly back into pyruvate via cardiac lactate dehydrogenase (LDH), which efficiently converts lactate back into pyruvate for entry into the citric acid cycle. During fasting or in the state of starvation, the heart consumes ketones to produce energy. While not a preferred source due to the need to process nitrogen, the heart can oxidize certain amino acids (especially branched-chain amino acids such as leucine, isoleucine, and valine) during extreme starvation or when other metabolic pathways are impaired. When CMs lose this remarkable metabolic flexibility, they undergo functional impairment and contribute to pathological cardiac remodeling. While mitochondria govern myocardial metabolic flexibility, the specific intra-mitochondrial and membrane-driven molecular mechanisms underlying this adaptation are outside the scope of this paper, and comprehensive reviews on these pathways can be found elsewhere [25,26].

**Table 1:** A comparison of fuel preference in fetal/neonatal, adult, senescent, and hiPSC-CM.

Cell Type	Main Substrate Use	Metabolic Profile	Key Features	Citations
Fetal/Neonatal CM	Glucose and lactate	More glycolytic	Immature mitochondria, lower oxidative metabolism	[27,28,29]
Adult CM	Fatty acids, some glucose, other fuels	More oxidative	High mitochondrial content, strong fatty-acid oxidation, exhibit metabolic flexibility	[15,16,21,30]
Senescent CM	Glucose	Shift towards more glycolytic	Loss of metabolic flexibility, mitochondrial impairment, increased inflammatory signaling	[31,32,33]
HiPSC-CM	Mostly glucose, low fatty-acid oxidation	Fetal-like, immature, more glycolytic	Reduced mitochondrial maturity, lower oxidative phosphorylation, limited flexibility	[14,34]

### 2.2. Neonatal/Fetal CM Metabolism

During cardiac development, the fetal and neonatal heart cells rely predominantly on glycolysis and lactate oxidation rather than fatty-acid oxidation. Although mitochondria are present, they are structurally immature and less efficient compared to those in the adult heart. This is not because they are defective, but rather because it is in an early developmental stage where the dominant substrate is glucose, and the metabolic shift to fatty-acid oxidation has not yet occurred [27]. This metabolic immaturity is characterized by high levels of Malonyl-CoA, which inhibits mitochondrial fatty acid uptake [28]. In addition, a recent study demonstrated that fetal sheep CMs exhibit lower fatty-acid oxidation rates and develop lipid droplets nearly 60% larger than those found in newborns [29]. This accumulation suggests that while the fetal heart is equipped for lipid uptake, it lacks the mature oxidative machinery required for efficient utilization.

Following birth, neonatal CMs gradually transition to utilizing fatty-acid oxidation to adapt to the high-fat content of the milk and oxygen-rich postnatal environment [27]. This metabolic shift following birth plays into the overarching trend in metabolic maturation. However, although fatty-acid-induced metabolic maturation is essential for adult-like function, recent evidence suggests that it acts as a double-edged sword, as CM proliferation is suppressed during this maturation process. Tanaka et al. demonstrated that treating neonatal rat CMs with a fatty acid mixture not only increased β-oxidation-related enzymes but also cell cycle arrest-associated factors such as pyruvate dehydrogenase kinase 4 (PDK4) and 3-hydroxy-3-methylglutaryl-coenzyme A synthase 2 [35].

### 2.3. Senescent CM Metabolism

Senescent CMs accumulate with age and acute injury, where they contribute directly to cardiac dysfunction [31,32,33]. Cellular senescence is defined as a stable cell cycle arrest in which cells lose the capacity to divide but remain metabolically active, with characteristic changes in gene expression, secretory profile, and metabolic state [36]. In proliferating cells, senescence is most often driven by the progressive telomere shortening with each division. CMs are largely terminally differentiated cells with turnover rates as low as 1% per year at age 20 and falling to 0.3% by age 75 [37]. Therefore, the canonical pathways that drive senescence are not entirely applicable to CMs. Even when telomeres are involved, the damage is length-independent and arises from persistent DNA damage signaling at telomeric foci rather than from telomere attrition [32]. Common senescence triggers in the heart include oxidative stress, ischemic injury, mitochondrial dysfunction, and chemotherapeutic agents such as doxorubicin (DOX) [38].

Following the onset of senescence, the metabolic profile of the CM shifts and both oxidative and glycolytic pathways become dysregulated. In addition to alterations in substrate utilization, senescent CMs exhibit impaired autophagic flux and mitophagy, which is the selective autophagic removal of damaged mitochondria [39]. Under physiological conditions, mitophagy maintains mitochondrial quality and preserves oxidative phosphorylation by eliminating dysfunctional mitochondria before they accumulate excessive reactive oxygen species (ROS). During cardiac aging and stress, however, suppression of mitophagy leads to the persistence of damaged mitochondria, increased oxidative stress, reduced ATP production, and activation of pro-senescent signaling pathways. Consequently, defective mitochondrial quality control further compromises the oxidative metabolic capacity of senescent CMs and amplifies metabolic dysfunction.

The first metabolic dysregulation mechanism involves direct lipotoxic damage to CM. In mice fed a high-fat diet as well as in hyperlipidemic human hearts, lipid overload suppresses sterol regulatory element-binding factor 2 (SREBF2), which in turn reduces the expression of the β-subunit of farnesyltransferase (FNTB). The resulting FNTB deficiency impairs maturation of nuclear lamin A, destabilizes the nuclear envelope, activates the DNA damage response, and drives CM senescence and downstream fibrosis. This was supported by a study that demonstrated that CM-specific overexpression of FNTB via adeno-associated virus rescues high-fat-diet-induced cardiac fibrosis, identifying a causal link between lipid stress and CM senescence [40].

The second failure occurs through glycolytic metabolism, which becomes activated under stress [15]. In induced cardiomyopathy, stabilized p53 not only drives CM senescence but also destabilizes hypoxia-inducible factor 1α (HIF-1α). This results in downregulation of GLUT1, GLUT4, and other glycolytic enzymes such as hexokinase 2, phosphofructokinase, pyruvate kinase, and lactate dehydrogenase, further decreasing the metabolic capabilities of the heart. Pharmacological inhibition of p53 with pifithrin-α then showed a reversal in the senescent phenotype and the glycolytic defects [41]. Taken together, these findings show that impaired mitophagy, lipid overload and stabilized p53 represent distinct but convergent routes from metabolic stress to CM senescence. Through these pathways, the cell’s ability to meet the high ATP demands required for cardiac contraction progressively reduces.

**Figure 1:**
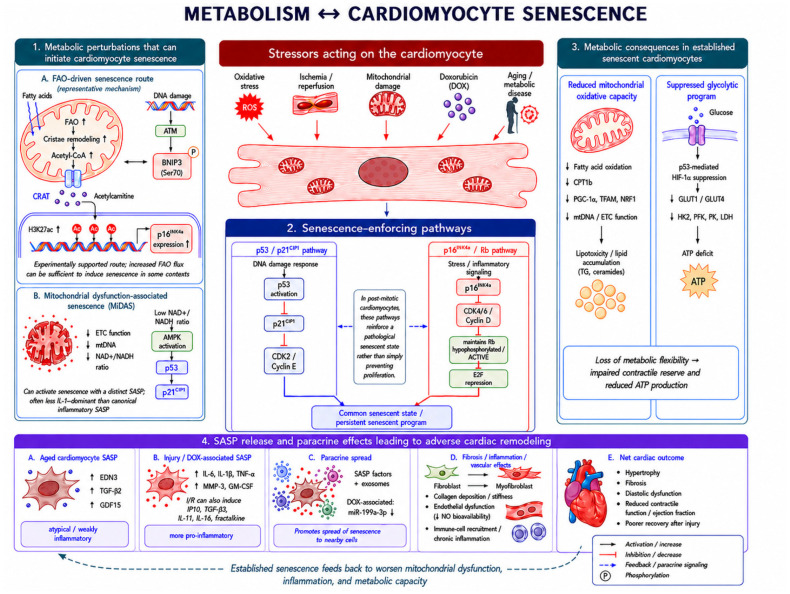
Metabolic dysregulation and CM senescence form a bidirectional, self-reinforcing cycle. Upstream stressors (oxidative stress, ischemia–reperfusion, mitochondrial damage, doxorubicin, aging) initiate CM senescence through two complementary mechanisms. 1(A) shows a FAO-driven route in which ATM phosphorylation of BNIP3 engages MICOS-mediated cristae remodeling and drives H3K27ac-dependent p16 activation. 1(B) shows the MiDAS route in which a decrease in NAD^+^/NADH activates AMPK and the p53/p21, leading to cell arrest. 2. Both p53/p21 and p16/Rb signaling pathways reenforce a pathological senescent state in post-mitotic CM. 3. Established senescent CMs show impaired mitochondrial oxidative metabolism, p53-mediated suppression of the glycolytic program, and loss of metabolic flexibility. 4. CMs release context-dependent SASP profiles (atypical EDN3/TGF-β2/GDF15 in aging; pro-inflammatory IL-6/IL-1β/TNF-α/MMP-3/GM-CSF after DOX; chemokine-heavy IP-10/TGF-β3/IL-11/IL-16/fractalkine after ischemia–reperfusion (I/R)), along with exosomes driving paracrine senescence, fibroblast activation, endothelial dysfunction, and adverse cardiac remodeling. The dashed feedback arrow indicates that established senescence further worsens mitochondrial dysfunction and metabolic capacity, completing the cycle.

The relationship between metabolic dysfunction and CM senescence is bidirectional, as illustrated in Figure 1. Recent works indicate that metabolic perturbation can initiate senescence rather than merely follow from it. Following DNA damage, mitochondrial remodeling is triggered. The ataxia telangiectasia mutated (ATM) kinase phosphorylates mitochondrial membrane protein BCL2 interacting protein 3 (BNIP3) at Ser70, which engages the mitochondrial contact site and cristae organizing system (MICOS) complex to accelerate fatty-acid oxidation. The resulting acetyl-CoA is then shuttled back to the nucleus to drive senescence [42]. In contrast, mitochondrial dysfunction-associated senescence (MiDAS) is triggered when mitochondrial respiration becomes impaired. Reduced electron transport chain capacity lowers the cytosolic NAD^+^/NADH ratio and activates AMP-activated protein kinase (AMPK), which in turn engages the p53/p21 to enforce cell cycle arrest [43]. Dietary supplementation with the polyamine spermidine in aged mice enhances autophagic and mitophagic activity, preserves CM mitochondrial respiration, and reverses age-associated structural and functional cardiac defects [44]. Notably, these benefits are completely abolished in mice with CM-specific deletion of the essential autophagy gene *Atg5*, demonstrating that intact autophagic machinery is required for protection against age-related cardiac decline [45]. Together, these findings identify that the BNIP3/MICOS/fatty-acid oxidation axis and the NAD^+^/AMPK/p53 pathway can both be manipulated to shift CM into and out of senescence. Importantly, these pathways are amenable to therapeutic intervention. For example, NAD^+^ supplementation has been shown to attenuate CM senescence and improve cardiac function in aged mice [46].

Upon undergoing cell cycle arrest, senescent CMs communicate their altered state to the surrounding microenvironment through profound structural and secretory transformations. Morphologically, these cells undergo hypertrophy and revert to a fetal-like gene expression program, upregulating structural and regulatory proteins characteristic of cardiac remodeling [32]. Simultaneously, senescent cells develop a complex Senescence-Associated Secretory Phenotype (SASP). Crucially, this secretory profile is not uniform but rather varies based on the specific stressor that induced senescence. While aged CMs exhibit no measurable difference in the classical pro-inflammatory cytokines such as interleukin-6 (IL-6), interleukin-1β (IL-1β), tumor necrosis factor-α (TNF-α), CXCL1, and CXCL2, they upregulate secreted factors such as endothelin-3 (Edn3), transforming growth factor β2 (TGF-β2), and growth/differentiation factor 15 (GDF15) [32]. On the other hand, DOX-treated CMs exhibit elevated matrix metalloproteinase-3 (MMP-3), IL-6, IL-1β, granulocyte–macrophage colony-stimulating factor (GM-CSF), and TNF-α [47]. Moreover, post-ischemic myocardium exhibits elevated interferon-γ-induced protein 10 (IP-10), TGF-β3, interleukin-11 (IL-11), interleukin-16 (IL-16), fractalkine, and IL-6 [48].

Rather than passively reflecting surrounding tissue inflammation, CMs are the primary drivers of this response. CM-specific deletion of senescence regulators drastically blunts downstream inflammatory signaling [33]. Ultimately, these localized secretory factors drive fibroblast-to-myofibroblast trans-differentiation, leading to massive collagen deposition [49] and destabilizing endothelial transcripts, which impairs nitric oxide production and vascular reactivity [50]. Additionally, senescence-associated chemokines such as IP-10 and MIP-3 actively recruit immune cells, establishing a chronic, damage-perpetuating inflammatory state. This chronic state is commonly referred to as inflammaging, a process that naturally accompanies biological aging [51]. Unlike the transient inflammation that resolves after acute injury, inflammaging is sustained by endogenous, sterile signals, including the SASP of senescent cells and the activation of innate immune sensors such as the NLRP3 inflammasome, whose activation is closely linked to CM senescence [52]. The cytokines in this state, particularly IL-6 and TNF-α, act directly on CM calcium handling machinery via sarcoplasmic reticulum Ca^2+^-ATPase downregulation [53]. Because senescent CM both arise within and feed back into this cycle, inflammaging establishes a self-reinforcing loop in which senescence drives inflammation and inflammation drives further senescence, exacerbating age-related myocardial decline.

The metabolic pathways detailed in this section offer a versatile framework for modulating metabolic function in both healthy and diseased adult myocardium. Furthermore, a deeper understanding of senescent CMs is highly relevant for disease modeling. Introducing controlled senescence-like phenotypes or aging-associated metabolic stressors to hiPSC-CMs can generate more physiologically relevant models for studying diabetic cardiomyopathy, heart failure, and age-related cardiac disorders.

### 2.4. Stem-Cell-Derived CM Metabolism

HiPSC-CMs have transformed cardiovascular research by offering a scalable and patient-specific cardiac cell source [54,55,56,57,58,59,60]. Despite their potential, immaturity limits the physiological relevance of hiPSC-CMs for modeling adult cardiac metabolism, disease progression, and drug responses. Studies have shown that hiPSC-CMs exhibit immature mitochondrial structures with high rates of glycolysis and low rates of fatty-acid oxidation that more closely resemble the neonatal hearts than adult myocardium [14,34]. These cells display sparse and disorganized mitochondria with a lower cristae density, which correlates with reduced basal and maximal oxygen consumption rates. During cardiac development, the transition to adult-like fatty-acid oxidation requires a synchronized perinatal burst of autophagy to actively degrade immature organelles before a mature mitochondrial network can expand [61,62]. However, hiPSC-CMs cultured in vitro exhibit immature and reduced autophagic capacity, which may contribute to their fetal-like phenotype [62].

Due to their predominant glycolytic metabolism, hiPSC-CMs exhibit an inherent resistance to necroptosis, which is a regulated form of cell death that combines features of apoptosis and necrosis, resulting in cellular swelling and membrane rupture [63]. Unlike adult CMs that are highly vulnerable to necroptosis during hypoxic stress, hiPSC-CMs utilize their fetal-like metabolic profile to survive low-oxygen conditions. Consequently, this metabolic resilience puts a limit on the use of hiPSC-CMs in accurately modeling ischemic injury or myocardial infarction [64]. A similar trend of lowered sensitivity to DOX due to immature mitochondrial function and a lower production of reactive oxygen species in comparison with adult CMs may also lead to an underestimation of cardiotoxicity risk in clinical settings [65]. Therefore, developing effective strategies to enhance metabolic maturation of hiPSC-CMs is essential for disease modeling and drug discovery. A comprehensive understanding of cardiac metabolism in both healthy and pathological states can reveal the molecular and metabolic mechanisms underlying CM maturation and guide the development of maturation strategies.

### 2.5. Metabolic Crosstalk Between CMs and Non-CMs

The heart is composed of CMs and non-CMs including cardiac fibroblasts, endothelial cells and immune cells that dynamically alter their metabolic programs in response to environmental cues [66]. While cardiac fibroblasts are not responsible for large production of cardiac ATP, they function as critical metabolic regulators of the heart. Cardiac fibroblasts modulate cardiac metabolism indirectly through extracellular matrix (ECM) remodeling, paracrine signaling and metabolic crosstalk. Following myocardial infarction or other cardiac insults, fibroblasts become activated and differentiate into a ‘myofibroblast’ phenotype [67]. Compared to quiescent fibroblasts, myofibroblasts are characterized by increased ECM production and a metabolic shift towards glucose and glutamine utilization to support proliferation and collagen synthesis [68]. The increased collagen density further results in limited oxygen and fatty-acid diffusion, thereby forcing CMs to undergo a metabolic shift from fatty-acid oxidation to glycolysis.

Beyond structural changes, fibroblasts influence heart health through the persistent secretion of paracrine signaling molecules and the release of specialized exosomes [69]. By discharging cytokines such as IL-6 and TGF-β, fibroblasts can directly impair mitochondrial respiration and trigger pathways that disrupt insulin sensitivity within the myocardium [70]. Highly glycolytic myofibroblasts also produce lactate as a byproduct and serve as metabolic donors [71]. Stressed CMs can then internalize and repurpose lactate as an alternative fuel source. This intimate exchange, facilitated by monocarboxylate transporters and exosomal transfer, proves that the heart’s overall metabolic efficiency and stress response are collaborative products of both its contractile and non-contractile cells. These biochemical signals act as a remote control for CM metabolism, altering gene expression and enzymatic activity without requiring direct physical contact between these two cell types.

While myofibroblasts demand more energy, their metabolic reprogramming is not solely driven by increased fuel intake. Interestingly, elevated extracellular glucose does not always translate to higher uptake, suggesting that glucose transporters may be operating at near-saturation [72]. Instead, the phenotypic shift is likely driven by intracellular metabolic signaling, where the presence of glucose triggers cellular changes regardless of how much substrate enters the cell [73].

Diabetic and hyperglycemic environments also affect cardiac fibroblast behavior. Studies showed that cardiac fibroblasts cultured under high glucose conditions exhibit decreased proliferative rate and increased collagen production [74,75]. On the contrary, other studies demonstrated increased proliferation of cardiac fibroblasts [76,77]. These differences may reflect cell source, species, matrix context, and passage number, all of which are important variables to consider when studying fibroblast metabolic responses.

In healthy hearts, endothelial cells supply CMs with oxygen, nutrients, and signaling molecules like nitric oxide and endothelin-1 [78]. In addition to passively delivering supplies, endothelial cells actively shape CM fuel selection, especially in diabetes. Endothelial cells ramp up lipid transport and energy metabolism programs, while CMs shift toward fatty-acid oxidation. Key signaling changes include Angptl4-Cdh5 and Angptl4-Sdc3 interactions, where endothelial-derived Angptl4 influences CM lipid handling [79]. High glucose damages endothelial function by disrupting fatty acid transporters and upregulating Angpt4, which limits the delivery of lipids to CMs. This consequently forces CMs into excessive fat-burning that can lead to lipotoxicity over time [80].

Macrophages make up most of the immune cell population, which contributes to 10% of non-CMs [81]. Macrophages are multi-functional immune cells that can polarize from a quiescent state into pro-inflammatory and anti-inflammatory phenotypes [82]. The fuel utilization is different in pro- and anti-inflammatory macrophages [83]. Pro-inflammatory macrophages primarily rely on glycolysis to support rapid energy production and biosynthesis of pro-inflammatory lipid mediators. Anti-inflammatory or reparative macrophages, on the other hand, favor oxidative metabolism, including mitochondrial oxidative phosphorylation and fatty-acid oxidation.

Resident macrophages play an important role in preserving metabolic stability of CMs by taking up dysfunctional mitochondria and other vesicles derived from CMs through the phagocytic receptor Mertk [84]. Upon injury, pro-inflammatory macrophages produce a variety of cytokines including TNF, IL-1, IL-6, IL-8, and IL-12 [85]. It has been reported that IL-1β and TNF-α are related to the increased expression of GLUT1 [86,87], which is the primary rate-limiting glucose transporter of pro-inflammatory macrophages [88]. Another study demonstrated that among all 13 known mouse GLUTs, GLUT1 is the only highly expressed GLUT by pro-inflammatory macrophages [89]. CMs also send out inflammatory cues, and activation of interferon regulatory factor 3 (IRF3) can induce type I interferons, chemokines, and genes associated with fibrosis. Recently, Kumari et al. demonstrated that this IRF3 activation in CMs causes impaired mitochondrial respiration and influences substrate use to shift away from efficient oxidative metabolism [90]. This suggests that energy failure and inflammatory signaling are tightly linked in CMs and dysfunctional CMs can amplify paracrine injury signals that reprogram fibroblasts, endothelial cells, and immune cells toward a more maladaptive state [79,90].

Beyond the intracardiac signaling, the heart is continuously influenced by endocrine input from systemic adipose tissue. Adipose-derived adipokines, including adiponectin, leptin, resistin, and visfatin, reach the myocardium through circulation and play important roles in CM fuel selection and mitochondrial function. Adiponectin activates AMP-activated protein kinase (AMPK) and peroxisome proliferator-activated receptor α (PPAR-α) signaling pathways, which promote fatty-acid oxidation [91,92]. Increased fatty oxidation enhances basal respiration and ATP production [93]. In contrast, leptin, resistin, and visfatin are considered pro-inflammatory adipokines. Each functions through an independent axis; however, the result is a pro-inflammatory response. Leptin engages the cardiac PI3K/mTOR/p70S6K pathway that drives hypertrophic growth [94] and resistin reduces insulin-stimulated glucose uptake in CMs, contributing to metabolic dysregulation [95]. Similarly, visfatin has been shown to induce CM hypertrophy and adverse remodeling [96]. In aging and metabolic disease, adipose tissue dysfunction alters the circulating adipokine profile and shifts the balance away from cardioprotective adiponectin and toward these pro-inflammatory mediators. This imbalance contributes to chronic inflammation, impaired metabolic homeostasis, and the development of senescent and pathological CM phenotypes.

## 3. Metabolism Remodeling in the Pathological Myocardium

**Figure 2:**
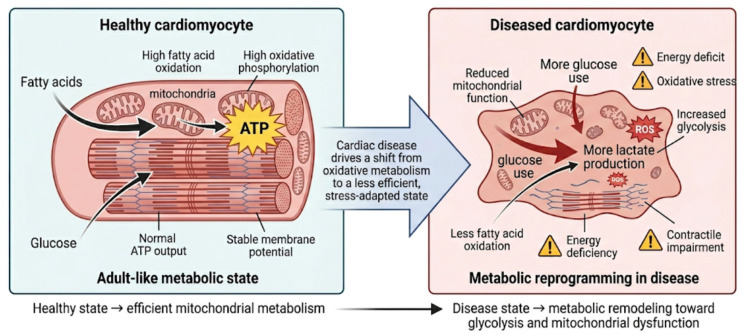
A schematic illustrating the metabolic shift from healthy to diseased CMs.

Under cardiac ischemia, restriction of blood flow creates a hypoxic environment that drives CMs to dynamically adapt their metabolism in response to environmental changes (Figure 2). Fatty acid utilization decreases due to the lack of oxygen supply, while glucose utilization increases as the citrate level decreases. Increased glucose uptake during cardiac ischemia may play a role in cardiac protection, as it is associated with improved myocardial function [97] as well as decreased mortality [98]. In an acute myocardial ischemia rat model, the heart attempts a protective shift by increasing the translocation of glucose transporters GLUT1 and GLUT4 to the sarcolemma to provide an oxygen-independent energy source to the ischemic tissue [99]. However, as the ischemia becomes more severe or prolonged, this compensatory mechanism fails. The lack of adequate perfusion prevents the clearance of metabolic byproducts, leading to an accumulation of intermediates that eventually inhibits further glucose utilization [100]. Consequently, while glucose metabolism spikes as an initial defense, it ultimately declines as the ischemic environment becomes increasingly toxic.

Cardiac hypertrophy is an adaptive response to increased workload caused by factors such as myocyte damage and hypertension [101]. Under hypertrophic conditions, the heart experiences elevated biosynthetic and energetic demands [9]. To accommodate these increased demands, CMs shift toward enhanced glucose uptake and glycolysis, accompanied by increased expression of GLUT1, GLUT3, and GLUT4 [8,102,103,104]. However, the role of glucose utilization appears to be complex and stage-dependent. Although many studies report increased glucose uptake during hypertrophy, a previous study using a mouse model of abdominal aortic constriction demonstrated that glucose utilization does not necessarily correlate with the severity of hypertrophy [105]. These findings suggest that the metabolic profile of the hypertrophied heart may vary depending on both the experimental model and the stage of disease progression. In addition to metabolic remodeling, cardiac hypertrophy is associated with other pathological changes, including the upregulation of structural genes such as *ACTA1* and *MLC2* [106], as well as mitochondrial dysfunction with reduced oxidative phosphorylation [9].

Similarly, in heart failure, alterations in the cardiac environment disrupt normal fuel utilization and drive metabolic remodeling of CMs. This process is marked by increased expression of GLUT1 and enhanced glucose uptake [107]. Despite this shift, pyruvate oxidation, which is a key step in glucose metabolism, is reduced, indicating impaired mitochondrial oxidative capacity. Mitochondrial dysfunction is further aggravated by elevated reactive oxygen species production, which can damage mitochondrial DNA, amplify oxidative stress, and, ultimately, lead to cellular injury. The increased glucose oxidation in failing hearts is mainly due to impaired fatty-acid oxidation and globally depressed maximal myocardial capacity to obtain energy from substrate [108].

During diabetic cardiomyopathy, the heart generally becomes less metabolically flexible and shifts towards greater reliance on fatty-acid oxidation. This metabolic remodeling is driven in part by upregulation of peroxisome proliferator-activated receptor-α (PPAR-α) signaling, which increases the expression of genes involved in fatty acid uptake and oxidation [109]. Diabetic hearts exhibit increased fatty acid uptake, largely mediated by the translocation and enhanced activity of the fatty acid transporter CD36 [110]. However, mitochondrial oxidative capacity is often insufficient to fully metabolize the excess lipid supply, resulting in intracellular accumulation of toxic lipid intermediates such as diacylglycerols and ceramides. The accumulation of lipotoxic intermediates is associated with a progressive decline in autophagic quality control as sustained diabetic stress suppresses both autophagic and mitophagic flux [111]. These lipotoxic species contribute to insulin resistance, mitochondrial injury, inflammation, and CM dysfunction [112]. Similar metabolic alterations are also observed in obesity-associated cardiomyopathy. In addition to altered substrate utilization, diabetic and obese hearts commonly develop mitochondrial dysfunction characterized by impaired oxidative phosphorylation, reduced metabolic efficiency, and excessive ROS production. Metabolic remodeling is additionally regulated at the post-translational level. In particular, reversible acetylation of mitochondrial and cytosolic metabolic enzymes has emerged as an important mechanism controlling substrate metabolism, mitochondrial function, and cardiac energy homeostasis in diabetes and obesity [112,113].

The chemotherapeutic agents used to treat cancer cause short- and long-term cardiotoxic effects, including arrhythmia, left ventricular dysfunction, heart failure, and other forms of cardiomyopathy. DOX is one of the most potent chemotherapy drugs, which also induces senescence in CMs. This chemotherapeutic exerts its effects through complementary mechanisms in the nucleus and mitochondria. DOX intercalates DNA and stabilizes the topoisomerase-II/DNA complex to induce double-strand breaks. In mitochondria, inhibition of the mitochondrial topoisomerase isoforms produces additional, persistent mitochondrial DNA damage that accelerates senescence [114]. In a Wistar rat model treated with DOX doses spanning the clinically relevant range, persistent mitochondrial DNA damage, mitochondrial dysfunction, and elevated left-ventricular senescence biomarkers were detectable at six to eight months post-injection. This aligns with the late-onset cardiotoxicity observed clinically in human patients receiving chemotherapy [114]. Because CMs depend almost entirely on aerobic metabolism, this mitochondrial injury is especially damaging to contractile function.

## 4. Modulators of Metabolic Maturation

### 4.1. Environmental Modulators

In addition to efforts to enhance structural maturation of stem-cell-derived CMs [115,116,117], recent studies have increasingly focused on achieving metabolic maturation, which remains a critical area requiring further investigation [64]. As 2D monoculture does not replicate the structural and cellular complexity of the native myocardium, 3D cultures have been widely considered to promote both structural and metabolic maturity in stem-cell-derived cells [118]. Instead of a traditional 2D monolayer differentiation, Correia et al. explored a 3D differentiation protocol in which hiPSC-derived cardiac progenitor cells were aggregated and differentiated under agitated conditions to generate hiPSC-CMs. They demonstrated that this 3D culture environment reproducibly enhanced the purity as well as metabolic maturation of hiPSC-CMs derived from different hiPSC lines [119]. The other study utilized a dissociation–reaggregation method on low-attachment surfaces to facilitate long-term 3D microtissue culture [120]. This format not only yielded viable, spontaneously beating tissues without a necrotic core, but also triggered a bioenergetic shift toward oxidative metabolism, exhibiting increased ATP utilization more closely mimicking an adult-like phenotype. Another study highlighted the importance of non-CMs by combining cardiac fibroblasts and endothelial cells into 3D microtissues, which resulted in improvement of structural as well as metabolic maturation of hiPSC-CMs [121]. Moreover, pre-treatment of hiPSCs with decellularized human cardiac ECM led to enhanced differentiation into hiPSC-CMs with higher functional maturity, including a more developed mitochondrial network, improved metabolic maturity, and a shift towards a more energetic profile [122]. Ulmer et al. also demonstrated that 3D-engineered heart tissue formed with hiPSC-CMs embedded in fibrin gel and cultured between elastic PDMS posts resulted in greater mitochondrial density and DNA content compared to cells in 2D culture [123].

Since CMs are continuously subjected to cyclic stretch, pressure, and shear forces that vary with physiological demand in vivo, the application of mechanical stimulation serves as one of the critical environmental cues that can promote both structural and metabolic maturity in the cells. One study investigated the effects of cyclical strain of varying magnitude ranging from 5% to 20% on hiPSC-CMs using their microdevice platform [124]. The authors demonstrated that while cyclical stimulation enhanced sarcomere organization and contractility of hiPSC-CMs, the effects are strain magnitude-dependent, and that optimization of mechanical stimulation is necessary to induce both structural and proteomic maturation. Another study demonstrated how hemodynamic forces can promote metabolic maturation using a microfluidic system [125]. HiPSC-CMs cultured under cyclic pulsatile flow exhibited enhanced cell alignment, contractility, sarcomere length, and mitochondrial network complexity. The improvement in mitochondrial complexity is especially relevant as it suggests that mechanical stimulation not only shapes tissue architecture, but also supports the mitochondrial remodeling needed for oxidative metabolism. In addition, a recent study has shown the direct relationship between the mechanical demand of the tissue and mitochondrial remodeling [126]. The authors demonstrated that dynamic cyclic shortening improves contractile function of engineered heart tissue, which generated a complete cardiac work loop compared to that under static isometric culture. Importantly, the regulation of mitochondrial protein expression was dependent on the amount of work done by the engineered heart tissue and not on the afterload.

Similar to mechanical stimulation, electrical stimulation has been recognized as one of the cues to induce maturation in CMs. Ronaldson-Bouchard et al. showed that engineered cardiac tissues subjected to progressively increasing electrical stimulation frequency (ranging from 2 to 6 Hz) and intensity over time displayed adult-like gene expression profiles with dense mitochondrial density and oxidative metabolism [127]. LaBarge et al. reported that cyclic uniaxial stretch combined with electrical stimulation improved structural maturation markers in hiPSC-CMs spheroids, including increased cTnI and MLC2v/a expression and improved Z-disk formation, while electrical pacing further enhanced conduction velocity, indirectly supporting metabolic maturation through increased functional workload [128]. A recent study examined the effects of lipid-enriched maturation medium combined with nanopatterning and electrostimulation on hiPSC-CMs [129]. The authors tested four groups, which included a control, metabolic medium (MM), MM + nanopatterning (NP), and MM + NP + electrical stimulation. After 42 days of culture, it was found that electrical stimulation is the critical driver promoting mitochondrial development, oxidative metabolic activity, and upregulation of genes associated with the electron transport chain and fatty-acid oxidation. Together, these studies indicate that electrical stimulation promotes not only structural maturation, but also mitochondrial remodeling and metabolic advancement in hiPSC-derived cardiac tissues. However, due to the varying stimulation timeframe, frequency, and pattern, there is limited data on the exact timeline on which hiPSC-CMs begin to exhibit measurable maturity when comparing stimulated vs. non-stimulated counterparts.

Despite substantial advancements in 3D in vitro tissue models, current platforms do not fully recapitulate the native myocardial niche since they usually lack functional perfusion and biochemical–mechanical feedback from ECM remodeling. Moreover, dynamic metabolic flexibility, intercellular substrate exchange, and mitochondrial quality-control mechanisms such as autophagy and mitophagy remain insufficiently characterized. Future studies incorporating metabolic flux analyses and more mature stromal and vascular cell populations are essential to reproduce the metabolic microenvironment of the adult heart.

### 4.2. Biochemical Modulators

One of the major determinants for a cell’s metabolic state is the nutrient composition in the culture media. Accordingly, modulation of nutrient composition in culture media is employed to promote metabolic maturation of hiPSC-CMs. In a recent study, researchers analyzed various components of media that contribute to hiPSC-CM maturation and long-term viability and identified that a galactose-based formulation containing creatine, carnitine, fatty acids, hormonal supplements, and taurine maximizes mitochondrial respiration while maintaining viability in comparison to other mixes [130]. They also demonstrated that by removing creatine, carnitine, and taurine, the levels of oxidative metabolism decrease while maintaining the same Ca^2+^ handling, which suggests that metabolic, functional, and transcriptomic maturation can be tuned separately.

Ramachandra et al. supplemented hiPSC-CMs with fatty acids for 30 days and observed improved mitochondrial remodeling, oxygen consumption rates, ATP synthesis, and enhanced structural features [131]. In contrast, 30 days with glucose exhibited suboptimal mitochondrial development and comparatively diminished bioenergetic function. This suggests that fatty acid metabolism-driven mitochondrial bioenergetics play an important role in promoting developmental maturation in hiPSC-CMs. Consistent findings were reported by another study, which demonstrated metabolic maturation of hiPSC-CMs with fatty acid treatment [132]. The authors differentiated hiPSC-CMs for 20 days, purified them in lactate-only medium and switched them to a fatty-acid-containing maturation medium. Upon assessing the function, fatty-acid-treated hiPSC-CMs had higher OCR for basal respiration, ATP production, maximal respiration, and spare respiratory capacity [132]. Finally, another study conducted by Querio et al. established that changing the culture substrate toward a fatty-acid-rich medium promoted hiPSC-CM maturation by shifting the cells away from a glycolytic fetal-like state and toward greater fatty acid utilization, oxidative metabolism, and adult-like structural organization [133]. In fact, researchers showed that hiPSC-CMs responded to insulin in a similar way in comparison to adult CMs, since they upregulated AKT/AS160 phosphorylation and translocated GLUT4 to the plasma membrane. These studies show that both fatty-acid supplementation and insulin-responsive glucose handling can support metabolic maturation in hiPSC-CMs and improve their usefulness for biomedical applications.

### 4.3. Molecular Modulators

Exosome-based therapies emerged as a promising cell-free alternative [134]. Exosomes are a subset of extracellular vehicles (EVs), typically 30–150 nanometers in diameter, formed via the inward budding of multivesicular bodies and released into the extracellular space upon fusion with the plasma membrane [135]. Exosomes are secreted by most cells in the heart, including cardiac and vascular cells [136]. Exosomes can deliver biologically active RNAs, proteins, lipids and other signaling molecules to recipient cells [135]. Recent studies demonstrated that exosomes derived from stem cells carry bioactive molecules, including microRNAs and proteins that can promote cardiac repair [134,137]. Exosomes secreted by a mixture of iPSC-derived CMs, endothelial cells, and smooth muscle cells have been shown to enhance myocardial bioenergetics and angiogenesis, while reducing cardiac hypertrophy, scar size, and cell apoptosis in a swine model [138]. Similarly, another study showed that exosomes isolated from the hiPSC-derived endothelial cells enhanced intracellular calcium transients, increased ATP content, and improved cell survival in a mouse MI model [139]. Recently, an in vitro study demonstrated that hiPSC-derived exosomes are effective in restoring mitochondrial function in Huntington’s disease neural stem cells [140]. Since CMs are highly dependent on mitochondria for proper functionality, this study presents the potential of exosome-based therapies broadly applicable for cardiac cells.

Another study demonstrated that the exosomes from hiPSC-derived MSCs regulate metabolic reprogramming of macrophages [141]. While senescent nucleus pulposus cells promoted macrophage polarization toward a pro-inflammatory phenotype, hiPSC-derived MSC-exosomes delivered miR-100-5p to macrophages, thereby suppressing mTORC1 signaling and glycolytic activity. This metabolic reprogramming ultimately shifted the macrophages toward a healing anti-inflammatory phenotype. A similar approach using exosome-based suppression of overactive glycolysis and mTORC1 signaling in CMs, particularly in the context of ischemic injury or heart failure, serves as a therapeutic intervention. In fact, co-culture of hiPSC-CMs with human mesenchymal stem cells (hMSCs) increased oxygen consumption, basal respiration rate, ATP production, and reduced ROS production [142]. In addition, delivery of exosomes derived from hiPSC-MSCs enriched in miR-9-5p to DOX-injured CMs attenuates senescence and mitochondrial fragmentation by suppressing the vascular peroxidase 1/extracellular signal-regulated kinase 1/2 (ERK1/2) signaling pathways [143]. These studies suggest that hMSC-derived soluble factors enhance mitochondrial energetics in hiPSC-CMs, further supporting the therapeutic potential of exosome therapies for damaged or senescing cells.

Taken together, the literature suggests that metabolic maturation of hiPSC-CMs does not follow a single, universal timeline. Rather, the rate and extent of maturation are highly dependent on the specific modality, and different maturation strategies achieve distinct metabolic endpoints. In general, early changes in respiration and substrate utilization are often detected within days to two weeks following biochemical or molecular interventions. For example, hiPSC-CMs cultured in maturation medium followed by metabolic medium exhibited enhanced respiratory function within 1–2 weeks, and an additional 12 days were identified as optimal based on functional readouts [130]. Exosome-mediated interventions induced measurable metabolic responses within approximately 24–72 h [142]. However, these studies primarily demonstrate acute metabolic enhancement rather than the establishment of a stable adult-like CM metabolic phenotype. In contrast, substantial mitochondrial remodeling, increased mitochondrial density, enhanced fatty-acid oxidation, and adult-like oxidative metabolism typically emerge over several weeks of prolonged fatty-acid exposure, 3D culture or electromechanical conditioning. Fatty-acid supplementation for 30 days induced greater mitochondrial remodeling and ATP production in hiPSC-CMs, while 30 days of glucose supplementation continued to harbor underdeveloped ultra-structural architecture and more subdued bioenergetics, constrained by suboptimal mitochondria development [131]. Similarly, improvements in maturation induced by mechanical or electromechanical conditioning typically require several weeks of culture. Such interventions, however, seem to refine metabolic and contractile processes rather than replace the need for prolonged culture in many systems [124]. Likewise, metabolic maturation in 3D tissues and microtissues is established over much longer timescales, ranging from 3 to 7 weeks in microtissues [120,121]. Combined electrostimulation and nanopatterning protocols demonstrated increased mitochondrial development, oxidative phosphorylation activity, and upregulation of fatty-acid oxidation pathways after approximately 42 days of culture [129]. These findings suggest that integrating multiple modalities may synergistically improve the efficiency and extent of CM maturation.

**Table 2:** A table summarizing various cues used in studies to achieve metabolic maturation in hiPSC-CMs.

	Maturation Cue	Description
**Environmental Modulators**	3D Tissue and ECM	3D differentiation using aggregate method results in enhanced purity and metabolic maturation of hiPSC-CMs from hiPSC-derived cardiac progenitor cells [119]. Long-term 3D microtissue culture triggered a bioenergetic shift in hiPSC-CMs toward oxidative metabolism, exhibiting increased ATP utilization [120]. 3D Tri-culture of hiPSC-CM, cardiac fibroblast and endothelial cells promotes both structural and metabolic maturation of hiPSC-CM [121]. Pre-treatment of hiPSC with decellularized human cardiac ECM resulted in enhanced differentiation into hiPSC-CM with a more developed mitochondrial network, improved metabolic maturity, and a shift towards a more energetic profile [122]. 3D engineered heart tissue formed with hiPSC-CM embedded in fibrin gel and cultured between elastic PDMS posts resulted in greater mitochondrial density and DNA content [123].
	Mechanical Stimulation	Cyclical strain ranging from 5% to 20% led to enhanced sarcomere organization and contractility of hiPSC-CM, although the resulting effects are strain magnitude-dependent [124]. HiPSC-CM cultured under cyclic pulsatile flow exhibited enhanced cell alignment, contractility, sarcomere length, and mitochondrial network complexity [125]. Dynamic cyclic shortening enhances metabolic maturation of hiPSC-CM in engineered heart tissue. The regulation of mitochondrial protein expression was dependent on the amount of work done by the engineered heart tissue [126].
	Electrical Stimulation	Progressively increasing electrical stimulation intensity resulted in greater mitochondrial development and coverage, oxidative metabolism, and upregulation of adult-like genes [127]. Cyclic uniaxial stretch combined with electrical stimulation improved structural maturation markers in hiPSC-CM spheroids, including increased cTnI and MLC2v/a expression and improved Z-disk formation, while electrical pacing further enhanced conduction velocity, indirectly supporting metabolic maturation through increased functional workload [128]. The combined effects of lipid-enriched maturation medium with nanopatterning and electrostimulation on hiPSC-CMs revealed that electrical stimulation is the critical driver promoting mitochondrial development, oxidative metabolic activity, and upregulation of genes associated with the electron transport chain and fatty-acid oxidation [129].
**Biochemical Modulators**	Metabolic Media	Galactose-based media formulation containing creatine, carnitine, fatty acids, hormonal supplements, and taurine maximizes mitochondrial respiration [130]. HiPSC-CMs in culture media supplemented with fatty acids for 30 days improved mitochondrial remodeling, oxygen consumption rates, ATP synthesis, and enhanced structural features [131]. Fatty acid treatment induces metabolic maturation of hiPSC-CMs [132]. HiPSC-CMs respond to insulin like adult CMs, making them a suitable model for cardio-metabolic research when analyzing membrane fractions rather than total lysates [133].
**Molecular Modulators**	Exosome-Based Therapies	HiPSC-derived exosomes are effective in restoring mitochondrial function in Huntington’s disease neural stem cells [140]. Exosomes from hiPSC-derived MSCs regulate metabolic reprogramming of macrophages [141]. Co-culture of hiPSC-CMs with hMSCs increased oxygen consumption, basal respiration rate, ATP production, and reduced ROS production [142]. HiPSC-MSCs enriched in miR-9-5p to DOX-injured CMs attenuates senescence and mitochondrial fragmentation [143].

## 5. Conclusions

Recent advances in the study of cardiac metabolism have elucidated important insights into the metabolic adaptations that support cardiac function in both physiological and pathological states. Numerous experimental and clinical studies have demonstrated that alterations in substrate utilization, mitochondrial function, and energy production contribute significantly to the development and progression of CVD. However, current evidence suggests that effective therapeutic modulation of cardiac metabolism will require integrative strategies that address both intrinsic metabolic dysfunction within CMs and the broader systemic and cellular environment influencing cardiac function.

A better understanding of senescent CMs can provide important insights for improving the metabolic maturation and therapeutic efficacy of hiPSC-CMs. Senescent CMs exhibit characteristic alterations in mitochondrial function, substrate utilization, oxidative stress, and cellular signaling that closely resemble features observed in aging and diseased myocardium. Understanding senescence-associated metabolic mechanisms can therefore guide strategies to design maturation protocols that promote healthy mitochondrial biogenesis, balanced substrate utilization, and improved oxidative capacity in hiPSC-CMs. For example, modulation of pathways involving PPAR-α, AMPK, PGC-1α, and Sirtuins may enhance mitochondrial maturation and metabolic flexibility while avoiding maladaptive lipid accumulation and oxidative injury. No single intervention is sufficient to induce full cardiac complexity and metabolic maturation in hiPSC-CMs. Further studies should explore the therapeutic potential of exosome-based therapies and identify the most effective combination of cues for promoting metabolic maturation. Investigating strategies that combine the metabolic enhancement of transplanted hiPSC-CMs with senescence protection of the recipient myocardium could reduce adverse remodeling and yield greater therapeutic efficacy.

## Data Availability

No new data were created or analyzed in this study.

## Abbreviations

The following abbreviations are used in this manuscript:

CVD	cardiovascular disease
CM	cardiomyocyte
hiPSC-CMs	human-induced pluripotent stem-cell-derived cardiomyocytes
CoA	acyl-coenzyme A
CPT-1	carnitine palmitoyl transferase 1
PDH	pyruvate dehydrogenase
SGLT1	sodium/glucose cotransporter 1
LDH	lactate dehydrogenase
MCT1	monocarboxylate transporters
PDK4	pyruvate dehydrogenase kinase 4
DOX	doxorubicin
SREBF2	sterol regulatory element-binding factor 2
FNTB	β-subunit of farnesyltransferase
HIF1-α	hypoxia-inducible factor 1 α
GLUT	glucose transporter
ATM	ataxia telangiectasia mutated
BNIP3	BCL2 interacting protein
H3K27ac	H3 lysine 27 acetylation
PPAR-α	peroxisome proliferator-activated receptor-α
AMPK	AMP-activated protein kinase
MIDAS	mitochondrial dysfunction-associated senescence
SASP	senescence-associated secretory phenotype
Il-6	interleukin-6
IL-1β	interleukin-1β
TNF-α	tumor necrosis factor-α
TGF β2	transforming growth factor β2
MMP3	matrix metalloproteinase-3
GM-CSF	granulocyte–macrophage colony-stimulating factor
MLC2	myosin light chain 2
ACTA1	actin alpha 1
MICOS	mitochondrial contact site and cristae organizing system
ECM	extracellular matrix
TCA	tricarboxylic acid
IRF3	interferon regulatory factor 3
EVs	extracellular vehicles
hMSC	human mesenchymal stem cells
ERK1/2	extracellular signal-regulated kinase 1/2
FAO	fatty-acid oxidation
GDF15	growth/differentiation factor 15
I/R	ischemia–reperfusion
ROS	reactive oxygen species
